# Traditional Chinese medicine compounds modulate signaling pathways to improve cardiac-related pathology

**DOI:** 10.3389/fphar.2025.1499060

**Published:** 2025-04-02

**Authors:** Luwen Zhang, Wei Wang, Xincan Liu, Kuipo Yan, Qiang Li, Ming Li, Chunying Li, Yanxin Li, Lei Chen

**Affiliations:** ^1^ The First Clinical Medical College of Henan University of Chinese Medicine, Zhengzhou, Henan, China; ^2^ The First Affiliated Hospital of Henan University of Chinese Medicine, Heart Center/National Regional (Traditional Chinese Medicine) Cardiovascular Diagnosis and Treatment Center, Zhengzhou, Henan, China; ^3^ The First Affiliated Hospital of Henan University of Chinese Medicine, Henan Province Traditional Chinese Medicine Epidemic Diseases Engineering Research Center, Zhengzhou, Henan, China; ^4^ The First Affiliated Hospital of Hena University of Chinese Medicine, Henan Key Laboratory of Viral Diseases Prevention and Treatment of Traditional Chinese Medicine, Zhengzhou, Henan, China

**Keywords:** cardiovascular disease, traditional Chinese medicine, nature compounds, signaling pathway, pharmacokinetic

## Abstract

Cardiovascular disease poses a significant risk to human health and remains the leading cause of illness and death globally, with its incidence continuing to rise. The intricate pathophysiological mechanisms of CVDs include inflammation, oxidative stress, autophagy, and myocardial fibrosis. In light of these underlying mechanisms, traditional Chinese medicine (TCM) and its constituents have demonstrated distinct advantages in managing CVDs. By exerting synergistic effects across multiple components and targets, traditional Chinese medicine can modulate the inflammatory response, mitigate oxidative stress, regulate excessive autophagy, and enhance myocardial fibrosis repair. This article reviews the latest advancements in understanding how TCM compounds regulate signaling pathways involved in the treatment of CVDs.

## 1 Introduction

CVDs pose a major global health challenge, as detailed in the latest World Health Organization report. Each year, these diseases result in over 17.9 million deaths, accounting for 31% of global mortality. Alarmingly, 75% of CVDs-related deaths occur in low- and middle-income countries (The WHO CVD Risk Chart Working Group, 2019). Projections suggest that by 2030, the annual death toll from CVDs could surpass 22.2 million. The increasing prevalence of CVDs, particularly among the elderly in these regions, exacerbates the burden on societal and economic systems, presenting a significant challenge for future healthcare systems (Mendis et al., 2015). The pathophysiological mechanisms underlying CVDs, such as myocardial fibrosis, autophagy, inflammation, and oxidative stress, may operate independently or synergistically to compromise cardiac structure and function, leading to conditions such as heart failure and myocardial infarction.

Herbal medicine, with a history spanning over 2,000 years, continues to play a vital role in clinical practice. Valued for their mild effects and low incidence of side effects, herbal medicine have gained significant interest and acceptance. However, the complex composition and inherent fluctuations of TCM can lead to variations in the content of active ingredients and pharmacological effects across different batches. For example, the content of salvianolic acid B in Danshen (Salvia miltiorrhiza) can vary depending on the origin, cultivation conditions, and processing methods. Therefore, rigorous quality control and standardized procedures are essential to ensuring the safety and efficacy of TCM. As modern medical research advances, the complex compounds within herbs and their diverse biological mechanisms there is an increasing understanding. These herbal treatments often demonstrate greater efficacy than conventional single-drug therapies, particularly in managing complex chronic conditions such as depression, schizophrenia, diabetes, and CVDs. This article aims to explore how herbs contribute to the treatment of CVDs.

## 2 Myocardial fibrosis

**FIGURE 1:**
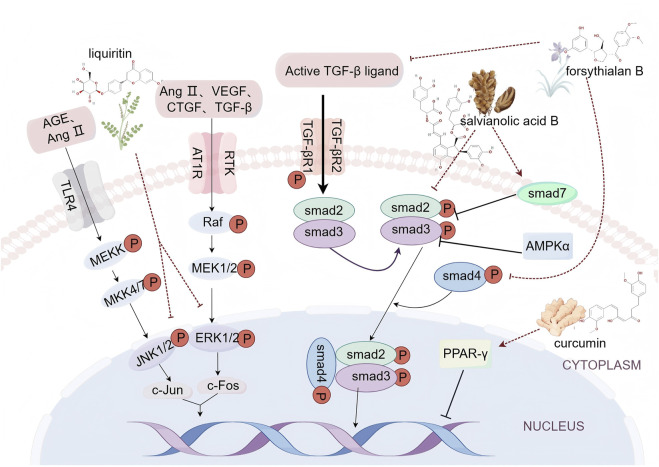
Schematic diagram of myocardial fibrosis signaling pathway (By Figdraw).

Myocardial fibrosis is a central pathological feature in various CVDs, including hypertension, myocardial infarction, and heart failure. It arises from an imbalance between collagen synthesis and degradation in cardiac fibroblasts (CFs), leading to excessive deposition of extracellular matrix proteins and an accumulation of activated CFs (López et al., 2021). Ischemic and hypoxic events, along with subsequent inflammatory responses and other stimuli, affect the myocardium, elevating levels of pro-fibrotic growth factors and cytokines both locally and in the circulatory system (Zhao et al., 2022). These growth factors and cytokines interact with their receptors, activating specific signaling pathways and transcription factors. Key signaling pathways associated with myocardial fibrosis include the TGF-β/Smad, mitogen-activated protein kinase (MAPK), and peroxisome proliferator-activated receptor gamma (PPAR-γ) pathways (Figure 1).

Among these, TGF-β ligand is a pivotal pro-fibrotic growth factor in myocardial fibrosis (Zhao et al., 2022). It modulates fibrosis through both classical and non-classical signaling pathways. In the classical pathway, TGF-β activates Smad proteins. The activated TGFβR1 initiates the phosphorylation of Smad2 and Smad3, which then form a complex with Smad4 and enter the nucleus to interact with other transcription factors. This interaction regulates the expression of target genes, promoting the synthesis of extracellular matrix proteins and contributing to the development and progression of myocardial fibrosis (Tzavlaki and Moustakas, 2020). Conversely, Smad7 acts as a negative regulator in the TGF-β/Smad signaling pathway. It inhibits Smad complex formation and function through mechanisms such as competitive binding to TGFβR1, promoting Smad protein ubiquitination and degradation, and modulating TGF-β pathway activity. This negative feedback mechanism is crucial for maintaining TGF-β signaling balance and regulating cellular processes such as limiting abnormal cell proliferation and fibrosis (Ma et al., 2018). TGF-β can also activate non-classical signaling pathways, notably the MAPK pathway. Abnormal activation of two key branches—extracellular signal-regulated kinases 1/2 (ERK1/2) and c-Jun N-terminal kinases 1/2 (JNK1/2)—fosters CFs proliferation and collagen maturation, thereby worsening myocardial fibrosis (Yousefi et al., 2020). Advanced glycation end products (AGEs) and angiotensin II (AngⅡ) stimulate the JNK1/2 pathway, increasing the expression of c-Jun protein, an oncogene product. Simultaneously, factors such as AngⅡ, vascular endothelial growth factor (VEGF), connective tissue growth factor (CTGF), and TGF-β activate the ERK1/2 pathway, which elevates the expression of c-Fos, another oncogene product. The dimerization of c-Jun and c-Fos proteins then enhances the expression of genes involved in CFs proliferation and collagen maturation (Zhang Z. et al., 2024). PPAR-γ, a member of the nuclear hormone receptor superfamily, is present in myocardial cells and plays a significant role in fibrotic responses linked to conditions such as hypertension, atherosclerosis, heart failure, and diabetic cardiomyopathy. Upon ligand stimulation, PPAR-γ binds to specific PPAR response elements (PPRE) within target genes, thereby regulating gene transcription (Liu H. J. et al., 2016). Recent research indicates that PPAR-γ activators reduce extracellular matrix (ECM) deposition and mitigate myocardial fibrosis, while the PPAR-γ antagonist GW9662 reverses these protective effects (Lu et al., 2021).

### 2.1 Salvianolic acid B

Salvia miltiorrhiza, commonly known as Danshen in TCM, is a perennial herb from the Lamiaceae family. It is renowned for its ability to enhance blood circulation, alleviate pain, and reduce inflammation. Danshen is widely used in the treatment of various circulatory system disorders (Xd et al., 2019). Salvianolic acid B, a highly water-soluble active compound in Danshen, has demonstrated cardioprotective effects against ischemia-reperfusion injuries in cardiomyocytes. Salvianolic acid B also reduces myocardial infarction size, improves cardiac function, and exhibits anti-fibrotic properties (Wu and Wang, 2012). In both *in vivo* and *in vitro* studies, it was revealed that ISO stimulation, the expression of pivotal signaling molecules TGF-β1 and Smad2/3 proteins within the TGF-β/Smads pathway was upregulated, whereas Smad7 expression was downregulated. Sal B administration markedly reversed this modulation (Gao et al., 2019). Further mechanistic exploration into Sal B’s ameliorative effects on diabetic cardiomyopathy-induced myocardial fibrosis demonstrated that Sal B significantly attenuated the ubiquitination of Smad7, thereby stabilizing Smad7 protein expression and subsequently inhibiting the TGF-β1 signaling cascade. Experimental findings indicated that following Sal B treatment, the expression of collagen I (Col-I), collagen III (Col-III), and α-SMA in murine myocardial tissue was significantly downregulated, suggesting that Sal B can diminish collagen secretion and inhibit cellular phenotypic transformation. Moreover, cell scratch assays and measurements of myocardial hydroxyproline concentration revealed that Sal B significantly suppressed the migratory capacity of high glucose-induced CFs and reduced their hydroxyproline secretion, further corroborating Sal B’s inhibitory effects on CFs migration and collagen secretion (Luo et al., 2023). This provides a scientific rationale for Sal B as a potential therapeutic agent for myocardial fibrosis.

As the primary active component of Danshen, Sal B exhibits diverse pharmacological activities. However, with its increasing clinical application, drug interaction issues have become more apparent, particularly in combination with cardiovascular drugs. Sal B can induce the activity of the CYP enzyme system, accelerating drug metabolism, reducing blood drug concentrations, and affecting efficacy. For instance, studies have shown that Sal B accelerates the metabolism of losartan by inducing the activity and expression of CYP3A4 and CYP2C9, thereby significantly reducing its Cmax (maximum plasma concentration), t1/2 (elimination half-life), AUC (area under the curve), and AUMC (area under the moment curve), thus shortening the duration of losartan’s pharmacological effects (Wang et al., 2016). *In vitro* experiments further confirmed that Sal B can induce the activity of CYP450 enzymes in rat liver microsomes, thereby accelerating losartan metabolism. Furthermore, molecular biology experiments and MTT assays revealed that Sal B dose-dependently induces the mRNA and protein expression of CYP3A4 and CYP2C9 in hepatocytes and has a significant inhibitory effect on hepatocyte activity at different concentrations (0.1–1,000 µM). This inhibition is both dose- and time-dependent, indicating that Sal B may have some toxicity to hepatocytes, especially at high concentrations and with prolonged exposure (Wang et al., 2016). Similarly, when Danshen is used in combination with atorvastatin, the main components of Danshen can induce the activity of CYP3A4 in liver microsomes, thereby affecting the metabolic stability of atorvastatin, shortening its half-life, and reducing its bioavailability (Sun et al., 2018). These interactions suggest that when used in combination with drugs such as losartan or atorvastatin, it may be necessary to adjust drug dosages to avoid reduced efficacy.

### 2.2 Forsythialan B

Callicarpa kwangtungensis, a member of the Verbenaceae family, is recognized for its significant therapeutic properties, including heat clearance, detoxification, promotion of circulation, and potent anti-inflammatory and antioxidant effects. Research has highlighted phenolic glycosides as the primary active components responsible for these effects. These glycosides serve as the pharmacological foundation of Callicarpa kwangtungensis, demonstrating efficacy in alleviating ischemic myocardial injury by reducing oxidative stress and apoptosis associated with inflammation (Sun et al., 2020). Forsythialan B, a phenylethanoid glycoside, has been identified in anti-fibrotic research. *In vivo* administration of forsythiaside B significantly ameliorates cardiac function and providing protection against myocardial fibrosis induced by isoproterenol (ISO). Forsythialan B impedes the activation of the TGF-β1 signaling pathway by suppressing the mRNA and protein expression of TGF-β1 in myocardial tissues and fibroblasts. Research indicates that Forsythialan Binhibits the phosphorylation and nuclear translocation of Smad3, and reduces the protein expression of Smad4, thereby weakening the transcriptional regulation of the Smad3/Smad4 complex on downstream fibrosis-related genes such as collagen III and α-SMA, effectively reducing the excessive deposition of extracellular matrix (Sun et al., 2021).

### 2.3 Liquiritin

Glycyrrhiza uralensis, commonly known as licorice, is a perennial herbaceous plant of the Fabaceae family. It is recognized for its medicinal properties, including moistening the lungs, alleviating cough, restoring qi, tonifying the middle-jiao, and exhibiting anti-tumor effects (Yang et al., 2015). Additionally, it has therapeutic applications in cardiovascular and cerebrovascular diseases. Liquiritin, a major bioactive compound in licorice, has shown cardioprotective effects. It alleviates stress-induced myocardial hypertrophy by modulating relevant signaling pathways. Liquiritin also regulates the proliferation and migration of vascular smooth muscle cells, enhancing its protective role in cardiovascular conditions, particularly those associated with coronary heart disease (Qin et al., 2022). In a high-fructose-induced myocardial fibrosis model, glycyrrhizic acid demonstrated anti-myocardial fibrosis effects through the integrated mechanisms of downregulating the expression of fibrosis-related genes, modulating oxidative stress, and suppressing inflammatory responses. Glycyrrhizic acid significantly inhibited the activation of the MAPK signaling pathway in high-fructose-induced CFs, suppressing the phosphorylation levels of ERK, p38 MAPK, and JNK, which subsequently reduced the expression of α-SMA in murine cardiac tissue and attenuated the overexpression of collagen I, collagen III, and MMP-9 induced by high fructose. Furthermore, glycyrrhizic acid mitigated the inflammatory response and reduced myocardial damage by downregulating the levels of activated IKKα, IκBα, and nuclear factor-κB (NF-κB), thereby reducing the production of mature IL-1β and inhibiting the expression of various inflammatory factors, including TNF-α, IL-6, IL-18, and IL-17. Regarding oxidative stress, high-fructose feeding led to decreased expression levels of the antioxidant enzymes SOD1 and SOD2 in murine hearts, whereas glycyrrhizic acid alleviated oxidative stress by upregulating the expression of these enzymes. Simultaneously, glycyrrhizic acid downregulated the expression levels of iNOS and TXNIP, which promote reactive oxygen species (ROS) generation, further mitigating oxidative stress (Zhang et al., 2016).

While Liquiritin is a primary constituent of licorice, the herb also contains glycyrrhetinic acid, liquiritin, and flavonoids. Given its complex composition, Glycyrrhiza uralensis may elicit various drug interactions and adverse effects when co-administered with other pharmaceuticals. Numerous studies have reported adverse reactions associated with Glycyrrhiza uralensis consumption, such as hypokalemia, hypertension, and metabolic alkalosis (Sabbadin et al., 2019; Bangert et al., 2021). Research suggests that glycyrrhizic acid, a major constituent of Glycyrrhiza uralensis, can inhibit 11β-hydroxysteroid dehydrogenase, thereby impeding cortisol metabolism and elevating mineralocorticoid levels, leading to apparent mineralocorticoid excess, which subsequently results in hypertension and hypokalemia (Ceccuzzi et al., 2023). Furthermore, glycyrrhizic acid may antagonize the effects of antihypertensive agents, diminishing their efficacy. For example, ACE inhibitors and ARB receptor antagonists exert their antihypertensive effects by reducing angiotensin II production, while the water and sodium retention and hypokalemia induced by glycyrrhizic acid can attenuate the antihypertensive effects of these drugs, as well as counteract the hypotensive effects of calcium channel blockers by increasing blood volume. Moreover, glycyrrhetinic acid and its derivatives can significantly inhibit 5-lipoxygenase, 12-lipoxygenase, and cyclooxygenase activity, thereby suppressing prostaglandin synthesis, inhibiting histamine synthesis and release, and reducing vasodilatory substances, leading to increased vascular resistance and, consequently, elevated blood pressure (Wu et al., 2024).

### 2.4 Curcumin

Curcuma longa, commonly known as turmeric, serves both culinary and medicinal purposes. Historical texts such as the *Bencao Gangmu* highlight its ability to prevent liver damage, reduce blood pressure and lipid levels, and alleviate pain, showcasing its significant therapeutic potential. Curcumin, the primary active compound in turmeric, is a natural polyphenol known for its effectiveness in treating various ailments (Kocaadam and Şanlier, 2017). Research demonstrates that curcumin can significantly inhibit angiotensin II-induced myocardial fibrosis, both *in vivo* and *in vitro*, through the upregulation of PPAR-γ. Elevated levels of angiotensin II lead to CFs proliferation, myocyte apoptosis, and excessive extracellular matrix deposition—critical processes in the development of myocardial fibrosis (Scalise et al., 2021). A study evaluating curcumin’s impact on myocardial fibrosis in spontaneously hypertensive rats, as well as its anti-fibrotic mechanism in rat CFs, shows that curcumin effectively downregulates collagen III and fibronectin in a dose-dependent manner following angiotensin II exposure. This effect is partially reversed by the PPAR-γ antagonist GW9662. Connective tissue growth factor (CTGF) and plasminogen activator inhibitor-1 (PAI-1) are identified as significant pro-fibrotic factors. In spontaneously hypertensive rats, CTGF and PAI-1 levels increase significantly in the left ventricle at both the mRNA and protein levels, indicating their involvement in hypertension-induced myocardial fibrosis. Moreover, curcumin enhances PPAR-γ activity, leading to substantial reductions in CTGF and PAI-1 expression in both *in vivo* and *in vitro* settings. These findings suggest that curcumin provides preventive and protective benefits against hypertension-related myocardial fibrosis (Meng et al., 2014).

Curcumin, the principal bioactive component of turmeric, exhibits a broad spectrum of biological activities, encompassing anti-inflammatory, antioxidant, and antineoplastic properties. Nevertheless, curcumin’s poor bioavailability leads to negligible plasma concentrations after oral administration, thereby hindering its capacity to attain therapeutic efficacy and consequently limiting its clinical utility. For example, the co-administration of curcumin with amlodipine, an antihypertensive agent, despite demonstrating significant vasodilation *in vitro*, failed to elicit hypotensive effects *in vivo*, potentially due to its suboptimal bioavailability (Alam et al., 2021). Furthermore, the safety profile of curcumin raises potential concerns, particularly with chronic administration and high-dose regimens. Research suggests that curcumin can induce histone hypoacetylation during early cardiac development, suppressing the expression of transcription factors such as GATA4, ultimately leading to cardiac developmental defects, including ventricular wall and septal thinning. Moreover, at elevated concentrations, curcumin increases intracellular reactive oxygen species levels and may potentially promote carcinogenesis (Sun et al., 2014). Curcumin also inhibits various drug-metabolizing enzymes, including cytochrome P450 and glutathione S-transferase, thereby affecting the metabolism of other drugs, leading to elevated plasma concentrations and potential toxicity (Kim et al., 2012). These adverse effects and drug interaction issues, to some extent, restrict the clinical application of curcumin.

Summary: Danshensu B, Forsythoside B, glycyrrhizin, and curcumin exhibit cardioprotective effects *via* multifaceted mechanisms. Extracellularly, Danshensu B and Forsythialan B attenuate the mRNA and protein expression of TGF-β1, decrease serum TGF-β1 levels, and impede its receptor binding. Glycyrrhizin suppresses the expression of oxidative stress-related proteins iNOS and TXNIP by reducing the secretion of inflammatory cytokines TNF-α, IL-6, and IL-18. Curcumin counteracts the pro-fibrotic effects of Ang II, thereby mitigating Ang II-induced ECM deposition. Intracellularly, Danshensu B reduces Smad2/3 phosphorylation, upregulates Smad7 expression, and inhibits its ubiquitination degradation, thus blocking the activation of the TGF-β1/Smads pathway. Forsythialan Balso inhibits Smad3 phosphorylation and Smad4 protein expression, preventing the formation of the Smad3/Smad4 complex. Glycyrrhizin inhibits the activation of the MAPK signaling pathway in the cytoplasm, suppressing the phosphorylation levels of ERK, p38 MAPK, and JNK, downregulating the activation levels of IKKα, IκBα, and NF-κB, and reducing the production of mature IL-1β. Curcumin inhibits downstream signal transduction of Ang II by activating PPAR-γ. At the stage of gene transcription regulation in the nucleus, the inhibition of Smad2/3 nuclear translocation by Danshensu B and the inhibition of Smad3/Smad4 complex nuclear localization by Forsythialan B both reduce the transcription of fibrosis-related genes such as Col-I, Col-III, and α-SMA. Glycyrrhizin inhibits the nuclear translocation of NF-κB, reducing the transcription of pro-inflammatory factors IL-1β, TNF-α, and MMP-9. Curcumin inhibits the gene expression of CTGF and PAI-1 through PPAR-γ, reducing the synthesis of Col-III and FN. These multi-target mechanisms of these compounds provide a scientific rationale for the prevention and treatment of myocardial fibrosis.

## 3 Autophagy

**FIGURE 2:**
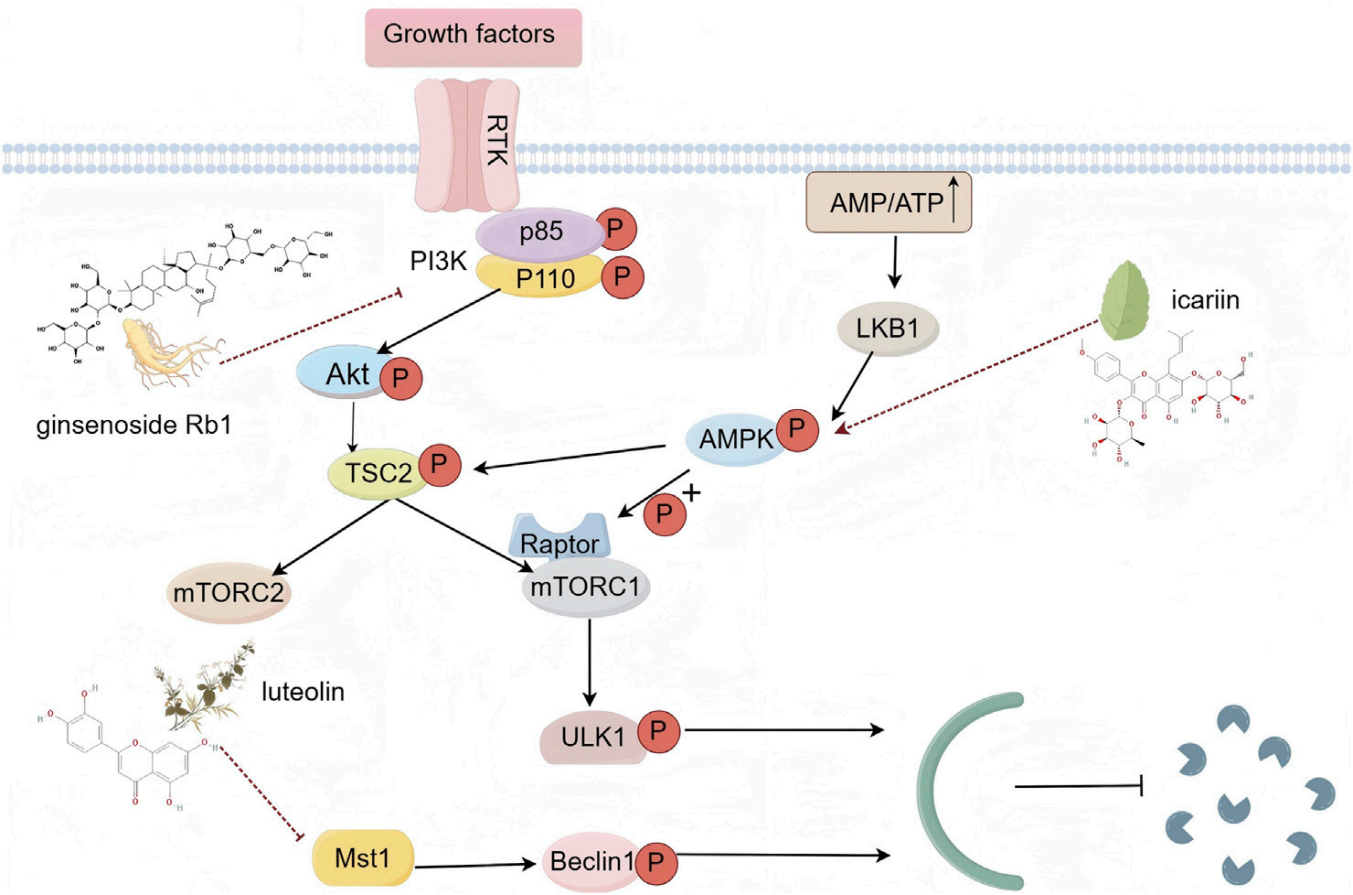
Schematic diagram of autophagy signaling pathway (By Figdraw).

Autophagy is a conserved lysosomal process that facilitates the degradation of cellular proteins and organelles, thereby maintaining cellular metabolic balance (Bu and Singh, 2021). It plays a critical role in various biological processes and is particularly relevant to the pathology of CVDs. As such, autophagy has emerged as a promising therapeutic target for these conditions. Additionally, autophagy affects cardiovascular health through several key signaling pathways (Gatica et al., 2022), such as the phosphatidylinositol-3-kinase (PI3K)/protein kinase B (Akt)/mammalian target of rapamycin (mTOR) pathway, the adenosine 5-monophosphate-activated protein kinase (AMPK)/mTOR pathway, and the mammalian sterile 20-like kinase 1 (MST1) pathway (Figure 2).

The PI3K/Akt/mTOR signaling pathway is fundamental in regulating autophagy. Inhibition of mTOR provides protection against myocardial cell injury. The PI3K heterodimer, comprising the regulatory subunit p85 and the catalytic subunit p110, is activated by growth factor receptors. This activation alters Akt protein structure, influencing the activity of various downstream substrates and playing a crucial role in regulating cell proliferation, differentiation, apoptosis, and migration. mTOR, a principal downstream target in the PI3K/Akt pathway, is a major focus of research in autophagy regulation. It exists in two distinct complexes: mTORC1 and mTORC2. mTORC1 primarily regulates cell growth, metabolism, and protein synthesis. Under nutrient-rich conditions, mTORC1 interacts directly with ULK1, a key protein for autophagy initiation, phosphorylating ULK1 at specific sites to inhibit autophagy initiation. Conversely, mTORC2 regulates cell survival, proliferation, and cytoskeletal organization. While it does not directly modulate autophagy, mTORC2 can influence it indirectly by phosphorylating downstream effectors such as Akt, which in turn affects mTORC1 activity. Under stress conditions, such as nutrient deprivation or hypoxia, the reduction in mTORC1 activity leads to the dephosphorylation and activation of ULK1, thereby triggering autophagy (Jiang et al., 2023; Sciarretta et al., 2018). AMPK is crucial for cellular energy regulation and signaling during autophagy (Wang S. et al., 2022). Stress-induced decreases in ATP levels and increases in the AMP/ATP ratio activate energy-sensitive kinases, particularly liver kinase B1 (LKB1), which acts as an upstream kinase for AMPK. Once activated, AMPK inhibits mTORC1 through two primary mechanisms, thereby influencing the autophagy process (Tamargo-Gómez and Mariño, 2018). MST1, a key component of the mammalian Hippo pathway, is a serine/threonine kinase that promotes cell apoptosis and inhibits compensatory myocardial hypertrophy, significantly impacting heart failure (Maejima et al., 2023). Studies have shown that MST1 regulates autophagy, cell death, proliferation, and organ size in response to pro-apoptotic signals. Specifically, MST1 inhibits autophagy by phosphorylating Beclin1 at threonine 108, which enhances its interaction with anti-apoptotic Bcl-2 family members. MST1’s phosphorylation of Beclin1 enhances its interaction with Bcl-2, impeding autophagy while promoting Bcl-2’s dissociation from Bax and triggering the apoptotic pathway (Maejima et al., 2013). Activation of MST1 during stress accelerates myocardial cell death by suppressing autophagy and promoting apoptosis. Conversely, inhibition of MST1 protects myocardial cells from cardiac stress-induced damage by stimulating autophagy and countering apoptosis (Maejima et al., 2013). A comprehensive understanding of these pathways enhances our knowledge of autophagy in cardiovascular health and disease. It also supports the potential use of TCM in modulating autophagy, providing a theoretical foundation for the development of novel therapeutic strategies.

### 3.1 Ginsenoside Rb1

Ginseng, a cornerstone of TCM, is renowned for its extensive pharmacological effects, including anti-cancer, anti-inflammatory, antioxidant, immune-modulatory, and neuroprotective properties (Park et al., 2012). Ginsenoside Rb1, a key active compound in ginseng, exhibits various protective activities within the cardiovascular system, including antioxidation, anti-apoptosis, and anti-arrhythmia effects (Zhou et al., 2019). Research indicates that ginsenoside Rb1 provides cardioprotective benefits by modulating the PI3K/Akt/mTOR signaling pathway. In studies focusing on myocardial ischemia-reperfusion injury (MIRI), ginsenoside Rb1 has been shown to significantly mitigate autophagy activation induced by MIRI. Immunohistochemical and Western blot analyses revealed increased expression of the autophagy-related protein Beclin1 in the MIRI group compared to the sham surgery cohort; however, treatment with ginsenoside Rb1 effectively counteracted this upregulation. Additionally, the levels of phosphorylated PI3K, Akt, and mTOR were significantly higher in the MIRI + Rb1 group compared to the MIRI group, which showed no changes in these levels. The cardioprotective effects of ginsenoside Rb1 were notably diminished when LY294002, a specific PI3K inhibitor, was administered, indicating that ginsenoside Rb1’s protective effects on myocardial function, both *in vitro* and *in vivo*, are largely dependent on the PI3K/Akt/mTOR signaling pathway. These findings suggest that ginsenoside Rb1 inhibits autophagy in myocardial cells and offers protection against MIRI through modulation of the PI3K/Akt/mTOR pathway (Qin et al., 2021).

Pharmacokinetic analysis in rats treated with a fixed-dose triple combination of non-valsartan/amlodipine/hydrochlorothiazide revealed that, in addition to its inherent pharmacological effects, ginsenosides may have potential herb-drug interactions with these agents. However, ginsenosides did not significantly alter the pharmacokinetics of hydrochlorothiazide, valsartan, or its metabolite, valsartan amide, with Cmax, AUC, T1/2, and mean residence time (MRT) remaining consistent whether administered alone or in combination with ginseng extract. Amlodipine was the only drug to exhibit pharmacokinetic changes, with a delayed Tmax observed after multiple doses of ginseng extract. This delay was attributed to ginsenosides reducing amlodipine’s intestinal permeability, thereby slowing absorption efficiency. Measurements of intestinal absorption rates showed a significant decrease in amlodipine absorption after repeated ginsenoside treatment, while valsartan and hydrochlorothiazide were unaffected (Jeon et al., 2022). Furthermore, studies have indicated that ginsenosides may induce furosemide resistance. Regular intake of ginsenosides in horses led to significant changes in furosemide pharmacokinetics, particularly an increased AUC and decreased clearance. These changes may be related to the inhibitory effects of ginsenosides on metabolic enzymes and efflux transporters. Although ginsenosides did not significantly affect the diuretic effect of furosemide, the alterations in its pharmacokinetics could still increase the risk of drug side effects. Therefore, patients undergoing treatment with furosemide or other loop diuretics should avoid using ginseng supplements (Kwak et al., 2023).

### 3.2 Icariin

Epimedium, classified as a mid-grade TCM in Shennong’s Classic of Materia Medica, is renowned for its significant medicinal properties. Icariin, a bioactive flavonoid derived from Epimedium, exhibits a range of pharmacological actions, including immune modulation, anti-inflammatory, antioxidant, and lipid-lowering effects (CVD, 2024). Recent studies have demonstrated that icariin can alleviate pathological myocardial hypertrophy by modulating autophagy. Research has shown that isoproterenol (ISO) inhibits autophagy, leading to the accumulation of misfolded proteins and damaged organelles, which ultimately impairs cardiac function. In experimental studies with rat models, icariin at a concentration of 20 µM effectively counteracted the reduction in H9c2 cell viability and the increase in cell volume induced by ISO. RT-qPCR and Western blot analyses revealed that icariin decreased the mRNA and protein levels of hypertrophy markers such as ANP, BNP, and β-MHC. Additionally, ISO treatment was found to reduce the phosphorylation of AMPK and its downstream autophagy-related proteins, Beclin-1 and Atg5, while increasing mTOR phosphorylation. Conversely, icariin administration effectively reversed these changes. The use of 3-methyladenine, a PI3K inhibitor, further intensified the effects of icariin. These findings suggest that icariin inhibits pathological myocardial hypertrophy primarily through the activation of AMPK and the inhibition of mTOR, thereby facilitating autophagy (Hu L. et al., 2022).

### 3.3 Luteolin

Luteolin, a flavonoid compound, is found in various vegetables, fruits, and TCMs such as carrots, peppers, apples, oranges, chrysanthemum, jasmine, and Danshen, primarily in the form of glycosides. This compound acts as a natural antioxidant and exhibits a range of biological activities, including antioxidation, anti-inflammation, anti-diabetes, and anti-cancer properties (Manzoor et al., 2019). Studies suggest that luteolin may reduce mortality associated with coronary artery disease and shows potential as a therapeutic agent for preventing and treating CVDs. Research indicates that luteolin can modulate autophagy, offering protective effects against various conditions (Hu et al., 2016). In a myocardial infarction (MI) model using Mst1 transgenic (Tg) and Mst1 knockout (Mst1^−/−^) mice, luteolin treatment enhanced autophagy in Mst1Tg mice experiencing MI but did not increase autophagy in Mst1^−/−^ myocardial cells. Additionally, in the luteolin pre-treatment group, autophagy flux improved in neonatal myocardial cells subjected to hypoxia, as evidenced by an increase in autophagosome puncta and a decrease in aggregate accumulation and P62 levels. Furthermore, luteolin increased mitochondrial membrane potential, ATP content, citrate synthase activity, and the activities of complexes I/II/III/IV/V in myocardial cells exposed to simulated MI injury. These findings highlight the cardioprotective properties of luteolin, attributing them to enhanced autophagy, reduced cell apoptosis, and improved mitochondrial biogenesis through Mst1 inhibition (Hu et al., 2016). Consequently, luteolin emerges as a promising therapeutic candidate for preventing and treating heart dysfunction and adverse cardiac remodeling induced by myocardial infarction.

Summary: Among the TCM compounds that modulate autophagy and related signals, ginsenoside Rb1, icariin, and luteolin primarily exert cardioprotective effects through intracellular mechanisms. Initially, within the cytoplasm, ginsenoside Rb1 significantly elevates the phosphorylation levels of PI3K and Akt, while inhibiting the expression of the autophagy-related protein Beclin1. Icariin promotes autophagy by activating AMPK and inhibiting mTOR activity. Luteolin can suppress Mst1, enhancing autophagic flux, leading to an increase in autophagosomes and a reduction in the accumulation of aggregates and P62. At the stage of gene transcription regulation within the nucleus, both ginsenoside Rb1 and icariin may regulate the transcription and expression of autophagy-related genes by modulating the phosphorylation level of the mTOR downstream transcription factor TFEB, although the specific mechanisms remain to be fully elucidated and require further investigation. The Mst1 inhibition by luteolin leads to dephosphorylation of the Hippo pathway downstream transcription factors YAP/TAZ, which then enter the nucleus to activate mitochondrial biogenesis-related genes, improving mitochondrial function and thereby mitigating myocardial infarction injury.

## 4 Inflammation

**FIGURE 3:**
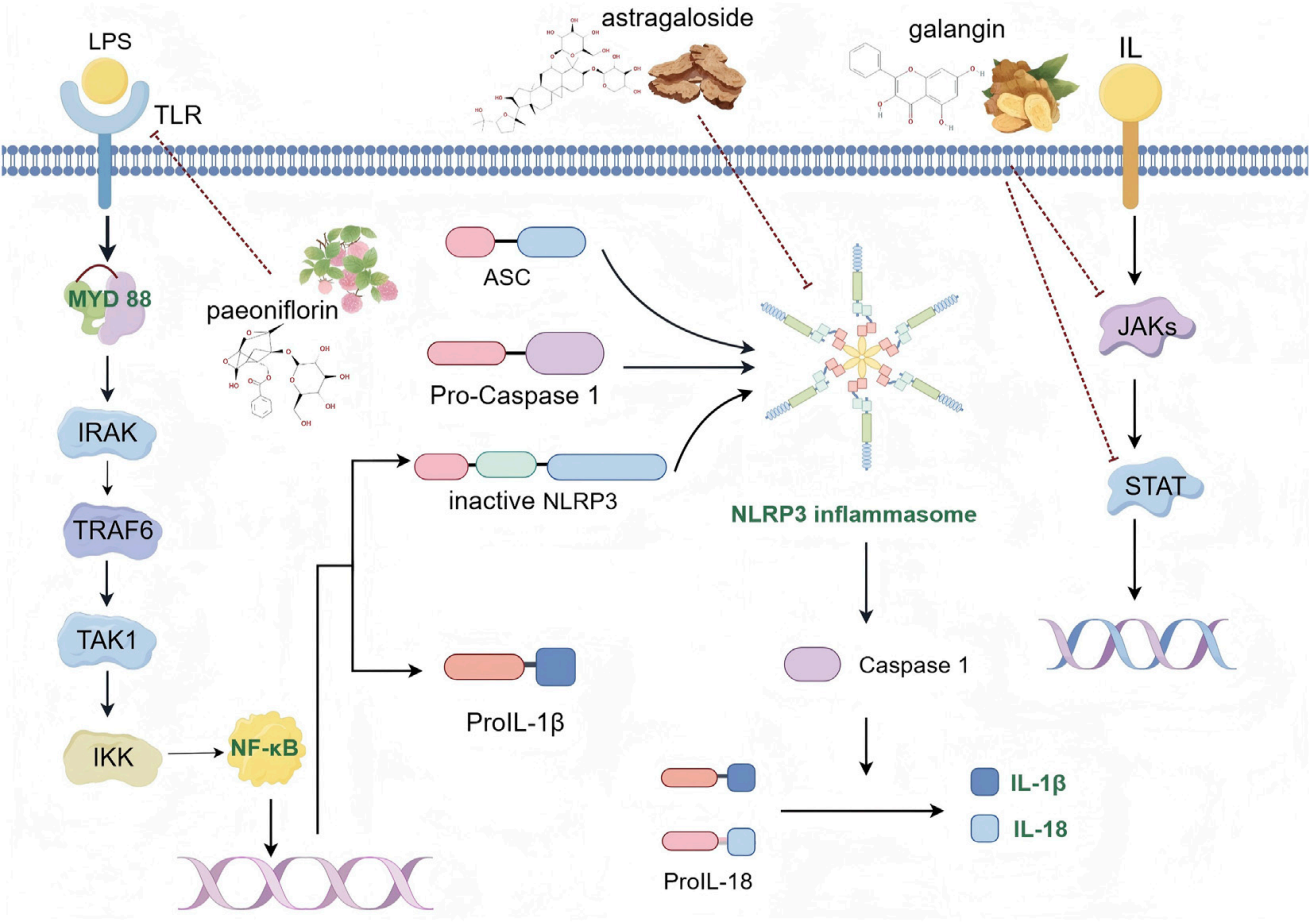
Schematic diagram of inflammatory signaling pathway (By Figdraw).

Inflammation is a crucial defense mechanism, representing the immune system’s response to harmful stimuli such as pathogen invasion, cellular damage, toxic exposure, and radiation. It plays a vital role in eliminating threats and initiating repair processes (Chen et al., 2018). However, increasing evidence highlights inflammation as a fundamental contributor to numerous CVDs, including atherosclerosis, myocardial infarction, arrhythmia, pericardial disease, valvular heart disease, myocarditis, and heart failure. Consequently, inhibiting early-stage cardiovascular inflammation can be advantageous for managing these conditions (Boyalla et al., 2023). The inflammatory response occurs in four key stages: 1) detection of harmful stimuli by cell surface pattern recognition receptors; 2) activation of inflammatory pathways (Chen et al., 2018); 3) secretion of inflammatory markers; and 4) recruitment of inflammatory cells. Several critical signaling pathways influence CVDs, including NF-κB, the NLRP3 inflammasome pathway, Janus kinase (JAK) signaling, and signal transducer and activator of transcription (STAT) pathways (Figure 3).

NF-κB, a pro-inflammatory transcription factor, has been extensively studied in macrophages, which are key components of the innate immune system and serve as the first line of defense against pathogens (Liu et al., 2017). Toll-like receptor (TLR) signaling, particularly through lipopolysaccharide (LPS), which is a ligand for TLR4, is crucial for modulating macrophage polarization towards the M1 phenotype. LPS activates intracellular signaling through two distinct TLR adapter proteins: myeloid differentiation primary response gene 88 (MyD88) and TIR domain-containing adaptor molecule inducing IFN-β (TRIF). The MyD88-dependent TLR pathway is essential for M1 macrophage polarization and the secretion of pro-inflammatory cytokines. This pathway activates IRAK kinase family members, which enhance the E3 ubiquitin-protein ligase activity of tumor necrosis factor receptor-associated factor 6 (TRAF6). TRAF6 undergoes self-ubiquitination and attaches ubiquitin chains to other signaling molecules, facilitating the activation of transforming growth factor-β-activated kinase 1 (TAK1). Activated TAK1 stimulates the downstream IKK kinase, leading to the phosphorylation and subsequent degradation of the inhibitor of NF-κB (IκBα), which in turn activates NF-κB (Kong et al., 2022). Activated NF-κB enhances the expression of pro-inflammatory cytokines and optimizes conditions for NLRP3 inflammasome activation. The NLRP3 inflammasome, a key regulator of immune and inflammatory responses, involves components such as the NLRP3 protein, the adaptor protein ASC (apoptosis-associated speck-like protein containing a CARD), and caspase-1 (Wang et al., 2020). The NLRP3 inflammasome activation occurs in two phases: initiation and protein complex assembly. The initiation phase is driven by pattern recognition receptors, notably TLR4 signaling, which activates the NF-κB pathway. This process promotes the transcription of NLRP3, interleukin-1β precursors (pro-IL-1β), and interleukin-18 precursors (pro-IL-18). Subsequently, NLRP3, ASC, and pro-caspase-1 assemble into a protein complex, leading to the cleavage of pro-caspase-1 into active caspase-1. Active caspase-1 then cleaves pro-inflammatory cytokines IL-1β and IL-18 into their active forms, thereby initiating and amplifying the inflammatory response (Paik et al., 2021). The JAK/STAT signaling pathway is also pivotal in the inflammatory response (Hu et al., 2021a). Inhibition of this pathway reduces inflammatory factors. Research shows that interleukin-12 (IL-12) activates the JAK/STAT4 pathway, which drives T cell differentiation into the Th1 phenotype. This differentiation promotes macrophage activation and the secretion of gamma-interferon (IFN-γ), IL-2, and TNF-α, which can exacerbate atherosclerosis and associated inflammation (Yang et al., 2020). These findings provide new insights and strategies for addressing inflammation-related CVDs, which remain prevalent and challenging due to the scarcity of effective drugs with minimal side effects in clinical practice. Various herbal compounds have demonstrated anti-inflammatory properties through mechanisms such as inhibiting the release of inflammatory mediators, regulating immune cell functions, and reducing oxidative stress at the cellular level.

### 4.1 Paeoniflorin

Paeoniae Radix, a traditional Chinese medicinal herb, is renowned for its analgesic, anti-inflammatory, antioxidant, and anticancer properties. It is particularly effective in addressing both cardiovascular and cerebrovascular disorders (He and Dai, 2011). The principal active compound in Paeoniae Radix, paeoniflorin, has been shown to offer substantial cardioprotective effects. Modern pharmacological research has demonstrated that paeoniflorin mitigates oxidative stress in myocardial cells, suppresses apoptosis, regulates autophagy, and modulates the NF-κB signaling pathway, thereby counteracting myocardial inflammation (Wu et al., 2020; Li et al., 2023). In the pathological mechanisms of CVDs, atherosclerosis, a common chronic inflammatory disease, is characterized by chronic inflammation of the arterial wall, accompanied by massive infiltration of macrophages and lymphocytes into the lesion. Experiments using an atherosclerosis rat model have shown that paeoniflorin treatment can significantly reduce serum total cholesterol, triglycerides, and low-density lipoprotein levels in atherosclerotic rats, while also improving the pathological state of the arterial intima, with effects comparable to the traditional lipid-lowering drug simvastatin. In the inflammatory response, the levels of inflammatory cytokines IL-1β, IL-6, and TNF-α were significantly elevated in the atherosclerosis group of rats, while paeoniflorin treatment significantly reduced the expression of these inflammatory cytokines. Through qPCR and ELISA detection, it was found that paeoniflorin inhibited the expression of TLR4 and MyD88, as well as the phosphorylation of IκBα and NF-κB p65. TLR4 plays a key role in initiating the innate immune response, which can lead to the activation of MyD88 and NF-κB, thereby promoting the production of inflammatory cytokines. Paeoniflorin alleviates the pathological process of atherosclerosis by inhibiting TLR4 and its downstream signaling pathways, thereby mitigating the inflammatory response (Wu et al., 2020).

Paeoniflorin not only mitigates the pathological progression of atherosclerosis but also demonstrates therapeutic efficacy in hypertension when combined with metoprolol. The co-administration of these two drugs at lower doses not only reduces blood pressure and improves microcirculation but also alleviates endothelial dysfunction by upregulating the expression of endothelial nitric oxide synthase (eNOS). Studies have shown that the combination of paeoniflorin and metoprolol results in a significant increase in Tmax, AUC, and MRT. Both paeoniflorin and metoprolol are substrates of CYP2D6, and they may interact to affect CYP metabolic enzyme activity, thereby enhancing the bioavailability of paeoniflorin (Li B. et al., 2018). However, the low oral bioavailability of paeoniflorin, coupled with limitations in its absorption and metabolism *in vivo*, hinders its widespread clinical application.

### 4.2 Astragaloside

Astragalus membranaceus is a perennial herb from the legume family, first documented in Shennong’s Classic of Materia Medica and widely recognized as one of the top TCMs for cardiovascular conditions (Liu et al., 2018). Clinically, it is valued for its qi-tonifying and blood-activating properties, which support normal blood circulation throughout the body. Astragaloside IV, a purified compound derived from Astragalus membranaceus, possesses a range of pharmacological effects, including anti-inflammatory, antioxidant, and anti-myocardial hypertrophy effects. Recent research highlights the significant advances in applying Astragaloside IV to CVDs (Shayganni et al., 2016; Yang et al., 2023b). In investigations of the effects of astragaloside IV on high-glucose-induced endothelial inflammation and its potential mechanisms, the NLRP3 inflammasome is activated under high-glucose conditions, leading to the release of inflammatory cytokines IL-1β and IL-18, thereby triggering an inflammatory response. The experimental results showed that the expression of NLRP3, ASC, caspase-1, IL-1β, and IL-18 were significantly reduced after astragaloside treatment. In addition, astragaloside also exerted anti-inflammatory effects by inhibiting the activity of TLR4/NF-κB pathway and CaSR. TLR4 is the upstream signaling molecule of NLRP3 inflammatory vesicle activation, and its activation promotes the activation of NLRP3 inflammatory vesicles through the NF-κB signaling pathway, and the study showed that astragaloside inhibited the activity of TLR4, which in turn blocked the phosphorylation of NF-κB as well as promoted the activation of NF-κB p65 translocation from the nucleus to the cytoplasm, attenuating the high glucose-induced inflammatory response in cardiomyocytes and thus exerting cardioprotective effects (Liu et al., 2018).

### 4.3 Galangin

Alpinia officinarum, commonly known as honey ginger or small good ginger, is the rhizome of the ginger plant and is documented in the *Pharmacopoeia of the People’s Republic of China*. It is characterized by its warm nature and pungent flavor, providing therapeutic effects such as warming the stomach, alleviating pain, and dispelling cold and wind. The primary active component of Alpinia officinarum is galangin, a natural flavonoid isolated from the root. Recent research has extensively documented the pharmacological activities of galangin, which include anti-inflammatory (Thapa et al., 2023), anti-tumor (Zhang et al., 2010), anti-fibrotic, and cardiovascular protective effects (Abukhalil et al., 2021), highlighting its potential as a therapeutic agent for various conditions. Galangin exerts multi-target anti-inflammatory and endothelial protective effects in ameliorating the progression of atherosclerosis by modulating the miR-124/STAT3 axis. Mechanistic studies revealed that galangin significantly upregulates the expression of miR-124 in vascular endothelial cells. Dual-luciferase reporter gene assays confirmed that miR-124 directly targets and binds to the 3′UTR region of STAT3, thereby blocking the mRNA translation and protein synthesis of STAT3. As a core effector molecule of the JAK2-STAT3 signaling pathway, the reduced phosphorylation level of STAT3 directly inhibits the transcriptional activity of downstream pro-inflammatory genes, such as IL-6, TNF-α, and IL-1β. Experimental results showed that the IL-6 level in the galangin-treated group decreased from 9.64 to 5.42 mg/L, and TNF-α decreased from 6.63 to 3.57 mg/L in the atherosclerosis model (Qi et al., 2025). Further studies found that the inhibition of STAT3 signaling not only reduces the inflammatory cascade by reducing NF-κB nuclear translocation but also inhibits endothelial cell apoptosis by regulating the Bcl-2/Bax protein ratio. These results suggest that galangin improves the progression of atherosclerosis and alleviates the inflammatory response by intervening in the JAK2-STAT3 signaling pathway through the miR-124/STAT3 axis (Yang et al., 2023).

Summary: The three compounds, paeoniflorin, astragaloside and galangin, synergize their anti-inflammatory effects through different signaling stages. Extracellularly, paeoniflorin and astragaloside target the TLR4 receptor, inhibit the expression of TLR4 receptor, and block the initiation of inflammatory signaling. In the cytoplasm, paeoniflorin downregulated the expression of MyD88 and inhibited the phosphorylation of IκBα, blocking the downstream signaling of TLR4; astragaloside directly bound to and inhibited the oligomerization of NLRP3 inflammatory vesicles, blocking their binding to ASC and caspase-1; and gorgonzine inhibited the translation of STAT3 and blocked the translation of JASC, through the upregulation of miR-124 and direct targeting to bind to the 3′UTR region of STAT3 mRNA. STAT3 translation, blocking the activation of JAK2/STAT3 signaling pathway. At the stage of gene transcription regulation in the nucleus, both paeoniflorin and astragaloside reduced the nuclear translocation of NF-κB p65 by inhibiting its phosphorylation, astragaloside was also able to inhibit NLRP3 inflammatory vesicles and indirectly reduce caspase-1-mediated release of IL-1β precursors; and gorgonzine inhibited the phosphorylation of STAT3 and reduced its nuclear translocation; these third compounds, through the above mechanisms, jointly reduce the synthesis of inflammatory factors such as IL-1β and IL-18.

## 5 Oxidative stress

**FIGURE 4:**
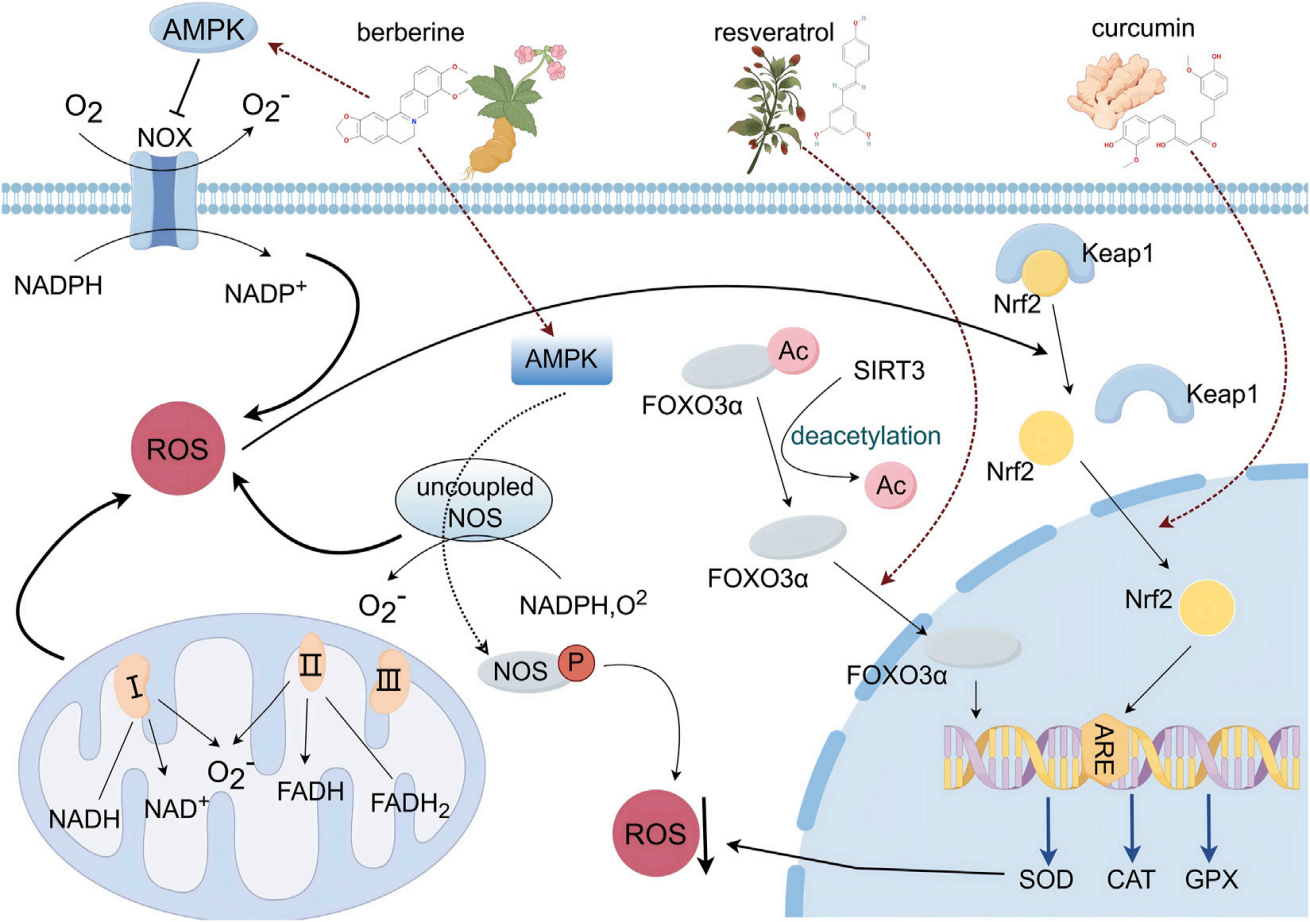
Schematic diagram of the oxidative stress signaling pathway (By Figdraw).

Oxidative stress refers to an imbalance between the production of ROS and the body’s antioxidant defenses. Maintaining this balance is crucial for normal cellular function. In cardiology, oxidative stress is closely linked to conditions such as myocardial infarction, ischemia/reperfusion injury, and heart failure (Pizzino et al., 2017). This imbalance is particularly evident in atherosclerosis and hypertension, where ROS generation exceeds the body’s capacity to neutralize them, leading to cell damage and death (Yan et al., 2023). Major sources of ROS include nicotinamide adenine dinucleotide phosphate oxidase (NADPH oxidase), the mitochondrial electron transport chain, and nitric oxide synthase (NOS), with approximately 90% of ROS generated in the cell originating from the mitochondrial respiratory chain (Dong et al., 2021). Endogenous antioxidants such as glutathione peroxidase (GPX), superoxide dismutase (SOD), and catalase (CAT) play a critical role in mitigating oxidative stress, especially in CVDs (Wang and Kang, 2020; Van et al., 2019). Compounds from TCM have shown significant efficacy in reducing oxidative stress and modulating key signaling pathways, including the SIRT3/FOXO3, AMPK, and Nrf2-Keap1 pathways (Figure 4).

The sirtuin family, including SIRT3, is essential for regulating cellular processes and protecting cells from oxidative damage (Santos et al., 2016). SIRT3, an NAD+ -dependent protein deacetylase, influences mitochondrial function and metabolism, particularly under stress conditions like caloric restriction. FOXO3, a forkhead transcription factor, is a key target of SIRT3. During oxidative stress, SIRT3 deacetylates FOXO3 at specific lysine residues, including K271 and K290, promoting its translocation to the nucleus. This enhances FOXO3’s transcriptional activity, which helps protect mitochondria from oxidative damage (Winnik et al., 2015). Increased expression of SIRT3 also reduces cellular ROS levels by activating SOD and bolstering antioxidant defenses, thereby protecting cardiomyocytes from oxidative stress-induced apoptosis. AMPK, a crucial cellular energy sensor, is involved in maintaining redox homeostasis (Shirwany and Zou, 2010). ROS can influence AMPK activity, which, in turn, regulates the expression of antioxidant defense genes and controls ROS production in the vascular wall. AMPK serves as a significant inhibitor of NADPH oxidases (NOX), which contribute to ROS overproduction and endothelial dysfunction (SONG and Zou, 2012). Mitochondrial uncoupling protein 2 (UCP2), located in the inner mitochondrial membrane, is part of the anion mitochondrial carrier family. UCP2-deficient mice exhibit increased oxidative stress and accelerated atherosclerotic plaque development. The transcription factor Nrf2 and its repressor, Keap1, are central to the cellular antioxidant response (Chen, 2021). Under normal conditions, Nrf2 binds to Keap1, which keeps Nrf2 activity inhibited (Yu and Xiao, 2021; Satta et al., 2017). However, oxidative stress causes Keap1 to undergo a conformational change, leading to the release of Nrf2. Once freed, Nrf2 translocates to the nucleus and binds to the antioxidant response element (ARE) within the promoter region of antioxidant enzyme genes. This interaction initiates the transcription of genes encoding key antioxidant enzymes, such as SOD and CAT, which work to reduce ROS levels and mitigate oxidative stress and associated cellular damage (Yu and Xiao, 2021; Hu et al., 2021b).

### 5.1 Resveratrol

Resveratrol is a non-flavonoid polyphenolic compound produced by various plants as a defense mechanism against environmental stressors. It is known for its significant anti-inflammatory and antioxidant properties. Natural sources of resveratrol include grape skins, blueberries, raspberries, mulberries, and peanuts (Pezzuto, 2019). Research has demonstrated that resveratrol may provide myocardial protection through the SIRT3/FOXO3a signaling pathway. Resveratrol enhances SIRT3 activity, thereby promoting the deacetylation of its downstream target, FOXO3a. Upon deacetylation, the FOXO3a transcription factor is activated and translocates to the nucleus, initiating the expression of intracellular SOD2 and CAT. This process reduces the accumulation of ROS during myocardial ischemia, thereby mitigating oxidative stress damage. Experimental findings demonstrate that obese mice administered daily resveratrol exhibited significantly increased nuclear translocation of FOXO3a and elevated antioxidant enzyme expression following myocardial infarction. Furthermore, experiments utilizing the selective SIRT3 inhibitor 3-TYP revealed that SIRT3 inhibition completely abrogated the cardioprotective effects of resveratrol. This observation further substantiates resveratrol as a natural and effective SIRT3 activator, capable of effectively reducing oxidative stress and myocardial damage induced by ischemia (Chen, 2021).

Resveratrol exhibits significant cardiovascular protective effects, demonstrating not only excellent oral tolerability and high bioavailability but also the capacity to enhance therapeutic efficacy when co-administered with other medications. Notably, resveratrol and captopril display synergistic effects in ameliorating cardiovascular damage induced by renovascular hypertension. In renovascular hypertension models, resveratrol significantly augments captopril’s vascular protective effects through its antioxidant properties, leading to reduced systolic blood pressure, decreased aortic remodeling and fibrosis. This is achieved by modulating nitric oxide production and inhibiting oxidative stress-mediated vascular smooth muscle cell proliferation (Natalin et al., 2016). Further studies indicate that resveratrol independently lowers blood pressure and demonstrates superior efficacy compared to captopril in mitigating myocardial hypertrophy and collagen deposition (Restini et al., 2022). Moreover, in rat models of myocardial infarction, resveratrol, either alone or in combination with valsartan, significantly reduces left ventricular dilation, improves ejection fraction, and lowers levels of oxidative stress markers and inflammatory cytokines. It also improves cardiac structure and function by inhibiting collagen deposition and reducing cardiac fibrosis (Raj et al., 2021). Despite resveratrol’s strong safety profile, concomitant use with warfarin increases the risk of bleeding, thus it is contraindicated in patients taking anticoagulants, antiplatelet agents, or nonsteroidal anti-inflammatory drugs (Huang et al., 2020).

### 5.2 Berberine

Coptis chinensis, first documented in Shennong’s Classic of Materia Medica, has long been known for its heat-clearing and detoxifying properties. Traditionally, it has been used to treat conditions such as ‘heart fire’ inflammation, palpitations, and insomnia (Pezzuto, 2019). Modern pharmacological research has shown that Rhizoma coptidis and its active constituents may be effective in addressing CVDs. Berberine, an isoquinoline alkaloid extracted from Rhizoma coptidis, is recognized for its therapeutic benefits, particularly in reducing the risk of CVDs (Feng et al., 2019). Its mechanisms of action include enhancing endothelial function, managing dyslipidemia, suppressing low-density lipoprotein oxidation, and lowering blood pressure (Affuso et al., 2010; Song et al., 2020). Research indicates that berberine primarily confers cardiovascular protection by modulating the AMPK signaling pathway and mitigating oxidative stress. In studies utilizing ApoE^−/−^ (apolipoprotein E-knockout) mice, berberine treatment correlated with increased uncoupling protein 2 (UCP2) expression and reduced markers of oxidative stress. Conversely, diminished UCP2 expression was associated with exacerbated inflammation and oxidative stress. In human umbilical vein endothelial cells (HUVECs), AMPK inactivation resulted in the abrogation of UCP2 mRNA and protein expression. As an AMPK activator, berberine treatment increased UCP2 mRNA levels by 2.4-fold compared to untreated controls. Furthermore, in ApoE^−/−^/AMPKα2^−/−^ mice, oxidative stress was augmented due to impaired UCP2 expression. The antioxidant tempol, a nitric oxide-based radical scavenger and spin trap, attenuated accelerated aortic lesions in ApoE^−/−^/AMPKα2^−/−^ mice, underscoring the role of reactive oxygen species (ROS) in lesion development. This suggests that berberine alleviates aortic lesions *in vivo* by activating AMPK and enhancing UCP2 expression, thereby inhibiting oxidative stress and vascular inflammation (Wang et al., 2011).

In the investigation of concomitant administration with losartan, the pharmacokinetic profiles of losartan and EXP3174 exhibited significant alterations in the presence of berberine. Elevated plasma concentrations of losartan and reduced plasma concentrations of EXP3174 were observed, consistent with the inhibition of losartan metabolism and decreased EXP3174 levels. These findings can be attributed to the inhibition of CYP3A4 or CYP2C9, given that losartan is a substrate of P-glycoprotein (P-gp) and is metabolized by CYP3A4 and CYP2C9 (Li et al., 2016). A two-phase, randomized, crossover clinical study in humans further corroborated the inhibitory effects of berberine on CYP2D6, CYP3A4, and CYP2C9, indicating a drug interaction between losartan and berberine. Within the context of drug interactions, investigating the effects of drugs on TCMs is equally crucial (Guo et al., 2012). For instance, the impact of irbesartan on the pharmacokinetics of berberine is noteworthy. Following oral administration of berberine in rats, its bioavailability is limited due to secondary absorption and enterohepatic circulation, resulting in rapid absorption and slow metabolism. Given that both compounds are lipophilic and interact with CYP3A4 and P-gp, co-administration of irbesartan enhances the bioavailability and absorption of berberine by inhibiting P-gp in the intestine, thereby prolonging its contact time with CYP3A4. Conversely, berberine increases irbesartan concentrations but has a minimal impact on irbesartan metabolism. Compared to individual oral administration, co-administration elevates the plasma concentrations of both berberine and irbesartan (Zhou et al., 2021).

### 5.3 Curcumin

Curcumin, the primary bioactive compound in turmeric, has been shown to possess potent antioxidant properties. Studies have shown that a concentration of 10 μM curcumin significantly reduces ROS levels in rat peritoneal macrophages. Curcumin functions as a dual-action antioxidant by directly scavenging ROS and indirectly triggering antioxidant responses. Further research highlights curcumin’s role in enhancing the activities of key antioxidant enzymes and activating the Nrf2-Keap1 signaling pathway, which plays a crucial role in promoting cellular survival. In experiments utilizing RAW264.7 cells as a research model, curcumin modulated Nrf2 activity, promoting its translocation from the cytoplasm to the nucleus and initiating gene transcription, thereby regulating the expression of antioxidant enzymes. All curcumin treatment groups exhibited increased CAT, SOD, and GPX activity. Low and medium doses of curcumin increased Nrf2 protein levels and facilitated its nuclear migration, significantly reducing intracellular ROS levels and mitigating oxidative stress (Pezzuto, 2019). Furthermore, co-administration of curcumin and metformin reduced oxidative stress damage through activation of the Nrf2/HO-1 pathway and alleviated inflammatory responses *via* inhibition of the JAK2/STAT3 pathway. The experimental results demonstrated that curcumin activated Nrf2, induced HO-1 expression, and enhanced cellular antioxidant capacity. By inhibiting the phosphorylation of JAK2 and STAT3, curcumin reduced the release of inflammatory factors such as TGF-β1 and IL-6, thereby mitigating inflammatory responses and exerting a protective effect on the myocardium (Zhu et al., 2024).

Summary: In the cytoplasm, resveratrol significantly upregulated SIRT3 expression. By enhancing SIRT3 activity, it promoted the deacetylation of the downstream protein FOXO3a. The cardioprotective effects of resveratrol were completely blocked by the selective SIRT3 inhibitor 3-TYP, indicating that resveratrol’s effects are dependent on SIRT3. Berberine, as an AMPK activator, upregulated AMPK activity, thereby promoting the expression of UCP2 mRNA and protein, inhibiting ROS production, and reducing inflammation and oxidative stress levels. Low/medium doses of curcumin significantly promoted the translocation of Nrf2 from the cytoplasm to the nucleus by modulating its activity, initiating gene transcription. During the gene transcription regulation phase in the nucleus, the deacetylated FOXO3a transcription factor translocated to the nucleus, initiating the expression of antioxidant enzymes SOD2 and CAT, reducing the accumulation of ROS during myocardial ischemia, and alleviating oxidative stress and myocardial damage. Curcumin significantly increased the expression of Nrf2 in the nucleus. After entering the nucleus, Nrf2 bound to antioxidant response elements, promoting the production of antioxidant enzymes CAT, SOD, and GPX, reducing intracellular ROS levels, and alleviating oxidative stress. In summary, these natural compounds exert cardioprotective effects via multiple mechanisms. Table 1 summarizes the mechanisms of action and signaling pathways of these compounds in the treatment of cardiovascular diseases.

**TABLE 1:** Mechanisms of action and signaling pathways of natural compounds in CVDs therapy.

Categorization	Compound name	Source (of information etc)	Related signaling pathways	Main mechanisms of action and results
Myocardial fibrosis	Salvianolic acid B	Salvia miltiorrhiza	TGF-β/Smad pathway	Reduces Smad2/3 protein expression, decreases ubiquitination level of Smad7, inhibits collagen I/III and α-SMA expression, and reduces collagen deposition
	Forsythialan B	Callicarpa kwangtungensis	TGF-β/Smad pathway	Inhibits Smad3 phosphorylation, downregulates Smad4 expression, reduces collagen synthesis, and significantly decreases TGF-β1 and α-SMA expression
	liquiritin	glycyrrhiza	MAPK pathway, NF-κB pathway	Inhibition of phosphorylation levels of ERK, p38 MAPK and JNK reduces collagen I/III and inflammatory factor expression and attenuates oxidative stress
	curcumin	turmeric	PPAR-γ pathway	Activation of PPAR-γ, inhibition of Ang II-induced fibrosis, and inhibition of CTGF and PAI-1 expression reduce ECM deposition
autophagy	Ginsenoside Rb1	ginseng	PI3K/Akt/mTOR pathway	Decreased expression of autophagy-related protein Beclin1 reduces myocardial injury
	Icariin	Epimedium, genus of herbaceous flowering plant, cultivated in the Far East as aphrodisiac	AMPK/mTOR pathway	Activates AMPK, inhibits mTOR, promotes autophagy, and inhibits cardiac hypertrophy
	luteolin	Carrots, peppers, honeysuckle, etc.	Mst1/Hippo pathway	Inhibition of Mst1, enhancement of autophagic flux, and improvement of mitochondrial function
inflammations	paeoniflorin	Paeoniae Radix	TLR4/MyD88/NF-κB pathway	Inhibition of the TLR4/NF-κB pathway and reduction of inflammatory factors IL-1β, IL-6, and TNF-α expression
	Astragaloside	Astragalus membranaceus	NLRP3 inflammatory vesicles, TLR4/NF-κB pathway	Inhibition of NLRP3 inflammasome activation, reduction of IL-1β and IL-18 expression, and inhibition of the TLR4/NF-κB pathway
	galangin	Alpinia officinarum	JAK2/STAT3 pathway, miR-124/STAT3 axis	Up-regulates miR-124 expression, inhibits JAK2/STAT3 signaling pathway, decreases IL-6 and TNF-α levels, and inhibits endothelial cell apoptosis
oxidative stress	resveratrol	Grapes, blueberries, etc.	SIRT3/FOXO3a pathway	Increased FOXO3a nuclear translocation and SOD2 and CAT expression and reduced ROS accumulation
	berberine	Coptis chinensis	AMPK/UCP2 pathway	Activates AMPK, increases UCP2 expression, and inhibits oxidative stress
	curcumin	turmeric	Nrf2-Keap1 access road	Activation of the Nrf2-Keap1 pathway increases CAT, SOD and GPX activities and decreases intracellular ROS levels

## 6 Discussion

Table 2 summarizes in detail the interventional roles of a variety of natural compounds in different CVDs pathophysiological mechanisms and the signaling pathways they target. These compounds exert interventional effects on CVDs-related mechanisms such as atherosclerosis, myocardial ischemic injury, myocardial hypertrophy, oxidative stress, and inflammatory responses, by regulating a variety of signaling pathways, including NF-κB, PI3K/Akt, Nrf2/HO-1, AMPK, and TLR4/NF-κB. The application of natural compounds in the treatment of CVDs has garnered significant attention, with clinical studies revealing substantial therapeutic potential while also highlighting critical issues that warrant further investigation. These studies typically employ randomized, double-blind, placebo-controlled trial designs. Some studies also incorporate multi-center designs to enhance the generalizability and representativeness of the findings. Sample sizes range from 50 to 300 participants, with follow-up durations varying from 4 weeks to 2 years. The research findings indicate that these natural compounds primarily exert their effects through anti-inflammatory, antioxidant, lipid-modulating, and metabolic-enhancing mechanisms. For instance, berberine has demonstrated notable efficacy in reducing the incidence of postoperative atrial fibrillation and improving cardiac function in patients with chronic congestive heart failure (Zeng et al., 2003; Zhang et al., 2022). Resveratrol has shown significant benefits in improving hemodynamic parameters, reducing erythrocyte aggregation, and ameliorating cardiac remodeling in hypertensive patients (Gal et al., 2020; Zheng et al., 2023; Magyar et al., 2012). Curcumin and its derivatives exhibit cardioprotective effects, including reducing the risk of myocardial infarction, improving endothelial function, modulating lipid profiles, and mitigating inflammation (Funamoto et al., 2016; Santos-Parker et al., 2017; Wongcharoen et al., 2012). However, these studies also present limitations. Bioavailability issues restrict the clinical efficacy of certain natural products, such as curcumin and resveratrol. Individual variability results in a lack of significant therapeutic effects in some patients. Moreover, most studies lack long-term follow-up data, which prevents a comprehensive assessment of the safety and potential side effects associated with the long-term use of these natural compounds and their supplements. Future research should further explore the long-term application value of these natural compounds and their potential synergistic effects when combined with other medications. Simultaneously, the development of personalized treatment strategies is essential to achieve synergistic effects and minimize adverse reactions, thereby facilitating improved clinical translation.

**TABLE 2:** Intervention and effect of natural compounds on pathophysiological mechanisms of CVDs.

Chemical compound	Pathophysiological mechanism	Targeting signaling pathways	Effect	Mechanism of action	
Salvianolic acid B	Atherosclerosis and the inflammatory response	NF-κB/NLRP3 signaling pathway	Lower blood lipid levels, reduce inflammation, reduce atherosclerotic plaque	Inhibition of NF-κB p65 nuclear translocation and NLRP3 inflammasome activation reduces expression of inflammatory factors (e.g., IL-1β, IL-18)	Zhao et al. (2023)
	Macrophage M1-type polarization and inflammation	Akt/mTOR signaling pathway	Reduction of pro-inflammatory factors (e.g., TNF-α, IL-6) and promotion of anti-inflammatory markers (e.g., IL-10)	Inhibits Akt/mTOR activation, promotes autophagy, and inhibits pro-inflammatory cell polarization	Zou et al. (2022)
	Myocardial ischemic injury and oxidative stress	SIRT1-PINK1-Parkin axis	Reduction of myocardial infarct size and improvement of electrocardiographic parameters	Promotes mitochondrial autophagy, maintains mitochondrial function, and inhibits NLRP3 inflammasome activation	Hu et al. (2020)
	Myocardial ischemic injury and inflammatory response	TLR4/NF-κB/NLRP3 signaling pathway	Improves heart function and reduces myocardial cell damage	Inhibition of TLR4/NF-κB activation, reduction of inflammatory factors (e.g., IL-1β), and inhibition of the initiation phase of NLRP3 inflammatory vesicles	Hu et al. (2019)
Forsythialan B	Macrophage inflammation and oxidative stress	JAK-STAT/p38 MAPK signaling pathway	Reduction of inflammatory factors, reduction of ROS accumulation	Inhibits JAK-STAT and p38 MAPK activation and reduces inflammatory factors (e.g., TNF-α, IL-1β)	Pan et al. (2014)
	Oxidative stress and apoptosis in cardiomyocytes	Nrf2/HO-1 signaling pathway	Increase cell viability and reduce the proportion of apoptotic cells	Activation of Nrf2/HO-1 signaling pathway enhances antioxidant capacity and restores mitochondrial membrane potential	Yang et al. (2022)
	Cardiac injury and inflammatory response induced by Kawasaki disease	SIRT1/NF-κB signaling pathway	Improvement of heart function and reduction of myocardial fiber disorders	Activation of SIRT1, inhibition of NF-κB p65 expression, reduction of cellular markers of focal death (e.g., caspase-1, NLRP3)	Yang et al. (2024)
Liquiritin	Fibrosis and inflammatory response after myocardial infarction	TLR4/MyD88/NF-κB signaling pathway	Improve heart function and reduce myocardial fibrosis	Inhibition of TLR4/MyD88/NF-κB activation and reduction of inflammatory factors (e.g., TNF-α, IL-1β)	Zhou et al. (2022)
	Pressure overload-induced myocardial hypertrophy and fibrosis	LKB1/AMPKα2/ACC signaling pathway	Improves cardiac function, reduces cardiomyocyte hypertrophy and apoptosis	Activation of LKB1/AMPKα2 signaling pathway and inhibition of mTORC1 phosphorylation	Aiyasiding et al. (2022)
Curcumin	Doxorubicin-induced cardiomyocyte scorched death and oxidative stress	PI3K/Akt/mTOR signaling pathway	Reduces serum cardiac muscle enzyme levels, reduces cardiomyocyte apoptosis	Activation of Akt/mTOR signaling pathway and inhibition of autophagy and focal death-related proteins (e.g., NLRP3, Caspase-1)	Yu et al. (2020)
	Diabetic cardiomyopathy and oxidative stress	Nrf2/HO-1 signaling pathway	Improves cardiac function, reduces myocardial fibrosis and lipid deposition	Activation of Nrf2/HO-1 signaling pathway reduces ROS generation and inhibits cardiomyocyte apoptosis	Wu B. et al. (2022)
	VSMC migration and inflammatory response in hypertensive rats	NF-κB signaling pathway	Lowers blood pressure, reduces aortic intima-media thickness	Inhibition of NF-κB activation and reduction of NLRP3 and MMP-9 expression	Han et al. (2019)
Ginsenoside Rb1	Heart failure and cardiomyocyte autophagy	Rho/ROCK pathway	Improvement of myocardial contractile function and reduction of cardiomyocyte apoptosis	Inhibits Rho/ROCK pathway, reduces ROCK protein expression, and inhibits autophagy	Yang et al. (2018)
	Atherosclerosis and oxidative stress	Keap1/Nrf2 signaling pathway	Reduces aortic plaque formation, reduces oxidative stress	Inhibition of Keap1, activation of Nrf2, reduction of oxidative stress and inflammatory response	Wang Z. C. et al. (2022b)
	Acute myocardial ischemia and mitochondrial damage	AMPKα/PINK1/Parkin mitochondrial autophagy pathway	Reduces myocardial infarct size and improves myocardial function	Activates AMPKα/PINK1/Parkin axis, promotes mitochondrial autophagy, and removes damaged mitochondria	Hu J.et al. (2022)
	Endothelial cell senescence and oxidative stress	Sirtuin-1/AMPK pathway	Protection of endothelial cells against oxidative stress and senescence	Activates Sirtuin-1, increases NAD^+^/NADH ratio, activates AMPK, inhibits cellular senescence	Zheng et al. (2020)
Icariin	Remodeling and fibrosis after myocardial infarction	TGF-β1/Smad signaling pathway	Increased left ventricular ejection fraction (EF), decreased myocardial fibrosis	Inhibition of TGF-β1 expression reduces Smad2/3 phosphorylation and attenuates myocardial fibrosis	Jia et al. (2023)
	Cardiac Aging and the Inflammatory Response	SIRT6/NF-κB signaling pathway		Upregulation of SIRT6 enzyme activity, inhibition of NF-κB (p65) nuclear translocation, and reduction of inflammatory factor expression	Chen et al. (2015)
Luteolin	Atherosclerosis and the inflammatory response	NF-κB signaling pathway	Reduces atherosclerotic plaque formation	Inhibition of the NF-κB signaling pathway reduces the expression of inflammatory factors and adhesion molecules	Jia et al. (2015)
	Myocardial ischemia-reperfusion injury	PI3K/Akt signaling pathway	Improvement of myocardial contractile function and reduction of cardiomyocyte apoptosis	Activation of the PI3K/Akt signaling pathway and increased expression of the anti-apoptotic protein Bcl-2	Fang et al. (2011)
	Atherosclerosis and disorders of lipid metabolism	AMPK-SIRT1 signaling pathway	Reduces atherosclerotic plaque formation and lipid accumulation	Activation of AMPK-SIRT1 signaling pathway reduces macrophage inflammation and lipid accumulation	Li J.et al. (2018)
Paeoniflorin	Cardiac Hypertrophy and Oxidative Stress	Nrf2/HO-1 signaling pathway	Reduce cardiac hypertrophy	Regulation of oxidative stress and inhibition of the Nrf2 signaling pathway	Ren et al. (2023)
	Chronic heart failure and myocardial remodeling	TGF-β1/Smad signaling pathway	Reduced myocardial remodeling	Inhibition of TGF-β1/Smad signaling pathway	Liu et al. (2019)
	Atrial fibrillation and atrial fibrosis	PI3K-Akt signaling pathway	Reducing Atrial Fibrosis	Inhibition of the PI3K-Akt pathway	Ji and Ning (2024)
	Sepsis-induced myocardial injury	p38 MAPK/NF-κB p65 signaling pathway	Reduces myocardial damage	Inhibition of the p38 MAPK/NF-κB p65 signaling pathway	Wang et al. (2021)
Astragaloside	Hypoxia-induced cardiac hypertrophy	CAPN1/mTOR signaling pathway	Reduce cardiac hypertrophy	Inhibits CAPN1 activation, activates mTOR signaling pathway, inhibits autophagy and apoptosis	Zhang et al. (2024b)
	cardiomyocyte apoptosis	TLR4/NF-κB signaling pathway	Inhibition of cardiomyocyte apoptosis	Inhibition of the TLR4/NF-κB signaling pathway	Zhao et al. (2017)
Galangin	Pressure overload-induced cardiac remodeling	PI3K-AKT-GSK3β signaling pathway	Reduces cardiac hypertrophy and fibrosis	Inhibition of the PI3K-AKT-GSK3β signaling pathway	Wu X. et al. (2022)
	Neoplastic endothelial formation after vascular injury	MEK1/2-ERK1/2-GATA4 signaling pathway	Inhibition of VSMCs proliferation and migration	Inhibition of MEK1/2-ERK1/2-GATA4 signaling pathway	Wang et al. (2019)
Resveratrol	cardiomegaly	NF-κB signaling pathway	Inhibits cardiomyocyte hypertrophy and fibrosis	Inhibition of NF-κB signaling pathway	Ma et al. (2023)
	Cardiac function and fibrosis after myocardial infarction	NLRP3 inflammasome/TGF-β1/SMAD2 signaling pathway	Improves heart function and reduces fibrosis	Inhibition of NLRP3 inflammasome and TGF-β1/SMAD2 signaling pathway	Jiang et al. (2021)
	Myocardial ischemia-reperfusion injury	TLR4/NF-κB signaling pathway	Reduced inflammatory response	Inhibition of the TLR4/NF-κB signaling pathway	Li et al. (2015)
	atherosclerosis	TLR4/NF-κB/HIF-1α signaling pathway	Suppression of the inflammatory response	Inhibition of the TLR4/NF-κB/HIF-1α signaling pathway	Guo et al. (2023)
	atherosclerosis	PI3K/AKT/mTOR signaling pathway	Lower lipid levels and reduce lesion size	Inhibition of PI3K/AKT/mTOR signaling pathway	Ji et al. (2022)
Berberine	Oxidized low-density lipoprotein-induced inflammatory response	AMPK/mTOR signaling pathway	Reducing the inflammatory response	Activates AMPK/mTOR signaling pathway and promotes autophagy	Fan et al. (2015)
	NLRP3 inflammasome-induced endothelial cell dysfunction	calcium signaling pathway	Protection of endothelial cell function	Inhibition of NLRP3 inflammasome activation and modulation of calcium signaling	Dai et al. (2022)
	carotid atherosclerosis	PI3K/AKT/mTOR signaling pathway	Reduces serum lipid levels and attenuates endothelial hyperplasia	Regulates PI3K/AKT/mTOR signaling pathway, promotes cell proliferation and inhibits apoptosis	Song and Chen (2021)

In the study of Chinese medicine compounds, pharmacokinetic properties and bioavailability are the key factors determining their clinical efficacy. According to their physicochemical properties, compounds can be classified into two categories, hydrophobic and hydrophilic, which have their own characteristics in bioavailability and pharmacokinetics. Hydrophobic compounds, such as curcumin, resveratrol, luteolin, berberine, and galangal, usually have high lipid solubility, which makes them less soluble in water, thus limiting their bioavailability. However, binding to plasma proteins such as lipoproteins can significantly increase the solubility and stability of the drug, improve its dispersion in body fluids, and prolong the duration of action of the drug in the body, thereby enhancing its bioavailability (Wasan et al., 2008). For example, curcumin has extremely poor water solubility (solubility of about 11 ng/mL), is difficult to dissolve in the gastrointestinal tract, has a very low absorption rate, and is susceptible to rapid conversion by metabolizing enzymes in the intestinal tract and the liver, making it difficult to maintain an effective blood concentration (Liu W. et al., 2016). To overcome these limitations, recent studies have been devoted to improving the bioavailability of curcumin. For example, curcumin can significantly improve its water solubility and stability by forming complexes with whey protein, casein or soy protein (Ye et al., 2021); its bioavailability can be significantly improved when used in combination with piperine (Lund and Pantuso, 2014); in addition, the use of nanoparticles, liposomes and other nanocarriers wrapped around curcumin can also significantly improve its solubility and targeting. Resveratrol, which also faces the problems of rapid metabolism and low bioavailability, can increase its peak plasma concentration by 1,000% by combining with piperine, which is mainly due to the inhibition of UDP glucuronosyltransferase activity and the reduction of glucuronide metabolite formation (Lund and Pantuso, 2014). In addition, the application of nanopreparations improves the stability and tissue absorption of resveratrol, resulting in increased concentrations in organs such as the brain, liver, and kidneys. The bioavailability of berberine is limited by first-pass metabolism, but its bioavailability and efficacy are expected to be improved by nanotechnology and structural modification. The oral bioavailability of lignocaine is relatively high because of its high lipid solubility, which can be improved by concomitant administration with lipids. Hydrophilic compounds, such as salvinorin B, coniferyl glycoside B, glycyrrhizin, ginsenoside Rb1, astragaloside, and paeoniflorin, usually have high water solubility, but their oral absorption may be limited, bioavailability is low, and they are prone to metabolic interactions with other drugs, which may affect the efficacy of the drug. For example, ginsenosides are susceptible to metabolism to secondary glycosides by intestinal flora due to their high molecular weight and hydrophilicity, resulting in low oral utilization; astragalosides have limited intestinal absorption due to their high hydrophilicity and poor membrane permeability, and are susceptible to exocytosis of P-glycoproteins, and need to rely on specific delivery systems (e.g., nanopreparations, phospholipid complexes) to enhance their cardiac targeting. The poor bioavailability of paeoniflorin due to its poor permeability, exocytosis by transporter proteins and hydrolytic degradation in the intestinal lumen further limits its clinical application (Liu et al., 2006). Instead, it was found that the bioavailability of paeoniflorin could be improved by blocking the efflux of the transporter proteins or by using glucosidase inhibitors. In addition, bioavailability can also be improved by structural modification of paeoniflorin, e.g., it has been shown that paeoniflorin-6′- O-benzenesulfonate and paeoniflorin-phospholipid complexes have higher bioavailability than paeoniflorin (Zhao et al., 2019). The bioavailability and pharmacokinetic properties of both hydrophobic and hydrophilic herbal compounds are challenging. In the future, new drug formulations or delivery modes need to be explored to improve the bioavailability of herbal compounds in order to fully utilize their potential therapeutic effects.

This review summarizes the beneficial therapeutic effects of traditional TCM and its active constituents in the treatment of CVDs. In TCM practice, multi-herb formulas are frequently employed, reflecting the multi-target characteristics of TCM therapy. In-depth investigation of the mechanisms of action of TCM components can further elucidate the essence of TCM treatment and provide structural guidance for the development of novel drugs. Therefore, a better understanding of the pathological mechanisms of CVDs and the pharmacological effects of natural drugs and active ingredients will facilitate large-scale clinical studies of these drugs and their targets in the future, helping us to gain a deeper understanding of the mechanisms of action of TCM in the treatment of CVDs and providing more precise directions for drug development and clinical treatment.
